# The Mangrove Nursery Paradigm Revisited: Otolith Stable Isotopes Support Nursery-to-Reef Movements by Indo-Pacific Fishes

**DOI:** 10.1371/journal.pone.0066320

**Published:** 2013-06-12

**Authors:** Ismael A. Kimirei, Ivan Nagelkerken, Yunus D. Mgaya, Chantal M. Huijbers

**Affiliations:** 1 Radboud University Nijmegen, Institute for Water and Wetland Research, Department of Animal Ecology and Ecophysiology, Nijmegen, The Netherlands; 2 Tanzania Fisheries Research Institute, Kigoma, Tanzania; 3 Southern Seas Ecology Laboratories, School of Earth and Environmental Sciences, The University of Adelaide, Adelaide, Australia; 4 College of Natural and Applied Sciences, Department of Aquatic Science and Fisheries, University of Dar es Salaam, Dar es Salaam, Tanzania; 5 Australian Rivers Institute – Coasts and Estuaries, Griffith University, Gold Coast campus, Southport, Australia; University of Canterbury, New Zealand

## Abstract

Mangroves and seagrass beds have long been perceived as important nurseries for many fish species. While there is growing evidence from the Western Atlantic that mangrove habitats are intricately connected to coral reefs through ontogenetic fish migrations, there is an ongoing debate of the value of these coastal ecosystems in the Indo-Pacific. The present study used natural tags, viz. otolith stable carbon and oxygen isotopes, to investigate for the first time the degree to which multiple tropical juvenile habitats subsidize coral reef fish populations in the Indo Pacific (Tanzania). Otoliths of three reef fish species (*Lethrinus harak*, *L. lentjan* and *Lutjanus fulviflamma*) were collected in mangrove, seagrass and coral reef habitats and analyzed for stable isotope ratios in the juvenile and adult otolith zones. δ^13^C signatures were significantly depleted in the juvenile compared to the adult zones, indicative of different habitat use through ontogeny. Maximum likelihood analysis identified that 82% of adult reef *L. harak* had resided in either mangrove (29%) or seagrass (53%) or reef (18%) habitats as juveniles. Of adult *L. fulviflamma* caught from offshore reefs, 99% had passed through mangroves habitats as juveniles. In contrast, *L. lentjan* adults originated predominantly from coral reefs (65–72%) as opposed to inshore vegetated habitats (28–35%). This study presents conclusive evidence for a nursery role of Indo-Pacific mangrove habitats for reef fish populations. It shows that intertidal habitats that are only temporarily available can form an important juvenile habitat for some species, and that reef fish populations are often replenished by multiple coastal habitats. Maintaining connectivity between inshore vegetated habitats and coral reefs, and conserving habitat mosaics rather than single nursery habitats, is a major priority for the sustainability of various Indo Pacific fish populations.

## Introduction

Coastal habitats such as mangroves and seagrass beds are acknowledged as important nursery habitats for various species of reef fish, most of which are important to fisheries [1], [2] and some of which are threatened [3]. These ecosystems are, however, highly affected by anthropogenic stressors like unsustainable fishing practices, habitat loss, and eutrophication [1], [4]. Seagrass beds are declining globally at rates of about 7% per year [5] while mangroves are decreasing in surface area by 1–2% per year [6]. Conservation and management of these habitats and their fisheries has received increasing attention based on their importance as juvenile fish habitat and their biological connectivity that enhances coastal marine productivity and biodiversity [7]. Likewise, designation and performance of marine protected areas (MPAs) can be improved by knowledge accrued from habitat connectivity studies [8], [9]. Because species that undergo ontogenetic habitat shifts cannot be conserved and managed by protecting single habitats, conservation efforts should focus on protecting habitat mosaics [10].

Until very recently, only indirect and circumstantial evidence existed in support of the paradigm that various species of coral reef fishes use mangroves or seagrass beds as essential juvenile habitat. Evidence was mostly based on higher juvenile densities and lower predation risk in these habitats as compared to the adult coral reef habitat (see review by [11]). Nurseries are defined as habitats whose ‘contribution per unit area to the production of individuals that recruit to adult populations is greater, on average, than production from other habitats in which juveniles occur’ [12]. Therefore, a habitat will only function as a productive nursery if its individuals reach adult populations, for which evidence of actual movement between habitats is of crucial importance. Long-term movement data to support ontogenetic cross-ecosystem shifts is difficult to obtain as artificial tags are expensive and not suitable for use in juvenile fishes or for long-term tracking. As a result, there has been an increasing focus on the use of natural tags such as stable isotope signatures in fish muscle tissue and earbones (otoliths) or elemental composition of otoliths [13].

The application of otolith chemistry to track fish movement is based on the assumption that fish living and feeding in different environments incorporate a detectable chemical tag if they reside in environments long enough [14]. Otoliths grow continuously throughout the life of a fish and remain chemically inert once formed, and can thus provide a detailed history of a fish's environment. The use of elemental chemistry is less suitable for non-estuarine tropical environments as the water chemistry of juvenile vs. adult marine habitats is usually more uniform [15] as opposed to those located along a gradient from fresh to marine waters in (temperate) estuarine regions. This problem does not arise when using stable isotope signatures of otoliths, such as ^12^C/^13^C ratios, which clearly differ among different vegetated habitats [16], [17]. Dissolved inorganic carbon (DIC) typically contributes 70–80% to otolith carbon and varies among water bodies around major vegetation types. Oxygen isotopes are often related to variability in water temperature and salinity and can thus provide a distinct signature of habitats in shallow, warmer water like mangroves and seagrass beds compared to coral reefs in cooler water [18]. Therefore, it is a very suitable method to determine ontogenetic shifts among habitats. Although otolith chemistry is recognized as a valuable tool, and has been increasingly used over the last decade, still very few studies have used otoliths to reconstruct the environmental history of fish [19]. Only very recently a few studies have provided convincing evidence of ontogenetic movement from Caribbean mangrove/seagrass nurseries to adult offshore habitat 20,21,22,23.

Studies on nursery function of tropical reef habitats have predominantly focused on the Caribbean region, while the much larger Indo-Pacific region remains largely unstudied [24]. There is no *a priori* reason to reject a potential importance of ecosystem connectivity for offshore productivity and replenishment of reef populations in the Indo-Pacific, and it is likely that coastal reef seascapes in the Indo-Pacific are connected in similar ways by fish movements as in the Caribbean [9], [24], [25]. The function of shallow-water ecosystems as juvenile habitat depends, however, on habitat availability and accessibility [26]. Unlike in the Caribbean where shallow-water habitats (especially mangroves) are permanently available to juvenile fish [27], Indo-Pacific mangrove systems along coastal shorelines are mostly available to fish only during high tides [9], [28]. Also, the arrangement of mangroves and seagrass beds in relation to reef habitats within the coastal seascape can profoundly affect the degree and type of ecological and biological connectivity between these habitats [29], [30]. In the Indo-Pacific region, clear-water vegetated habitats are often more intermixed compared to Caribbean islands where they are spatially separated, while the much larger tidal ranges in the Indo-Pacific facilitate non-ontogenetic reef fish movements [28]. The large tidal range potentially also leads to stable isotope signatures showing more overlap among different habitats due to tidal exchange of water bodies between habitats [31], [32].

Even though the Indo-Pacific region harbors vast areas of mangroves and tropical seagrass beds, our understanding of the nursery role of these habitats in this region remains rudimentary. Based on fish density data, there has been a long standing debate of this function in this region (see reviews by [33] and [11]). The consensus from most studies is that Indo-Pacific mangroves play a minor role as critical juvenile habitat for reef or offshore fish species [34], [35], [36]. Nagelkerken [24] argued, however, that the apparent disparity between the two biogeographic regions is based on an invalid comparison, confounded by differences in tidal range (low vs. high), salinity (estuarine vs. marine), and spatial setting (island vs. continental coastlines). Another questionable argument that has previously been used to evaluate nursery function is that relatively few Indo-Pacific fish species appear to depend on mangrove habitats [36]. However, if these few species are of high commercial importance, are highly abundant species, or fulfill important ecological roles, then they can have important impacts on ecosystem production, functioning and resilience [11], [37], [38].

Recent studies have found that during their juvenile stage multiple species are present only in Indo-Pacific mangroves [39], [40], [41], and that isolated reefs show significantly lower adult densities of such species than reefs directly adjacent to these ecosystems [42]. This suggests that mangroves in this region could play a valuable role in replenishing offshore populations of some species. Only a couple of studies have provided unambiguous evidence of nursery-to-reef movement in the Indo-Pacific [17], [21], but failed to separate the role of individual juvenile habitats (e.g. mangrove vs. seagrass vs. reef). As a result, we know little of how different nursery habitats contribute to overall replenishment of offshore adult populations. This gap of knowledge is concerning as species may show different dependencies on different nursery habitats, and this dependency may further change through ontogeny. Hence, identifying the individual contribution of multiple nursery habitats to adult populations is of critical importance for management and conservation purposes. The objective of this study, therefore, was to test to which degree a suite of Indo-Pacific reef fish species has passed through putative mangrove vs. seagrass vs. coral reef nursery habitat. We analyzed a robust dataset of otoliths from several Indo-Pacific coral reef fish species collected at nearshore and offshore reef sites in Tanzania. Our results reveal the degree of connectivity among Indo-Pacific tropical coastal habitats, and we evaluate the role of two critical, circumtropical juvenile habitats for adult reef fish populations at different distances from the shore.

## Materials and Methods

### Ethics Statement

This study was not evaluated by an animal ethics committee because there was no such committee in Tanzania during the course of the study. We obtained written permission from the Director of Fisheries in the Ministry of Livestock and Fisheries Development (MLFD) of the United Republic of Tanzania (URT) in 2009 [43] to capture fish on the reef using spear guns and fish in the mangroves using nets. On the coral reef, fish were sacrificed under water directly after spearing by cervical dislocation. Mangrove and seagrass fish were mostly supplied dead by fishermen, but the fish we caught ourselves in the mangroves were sacrificed by hypothermia.

### Study Area and Species

**Figure 1:**
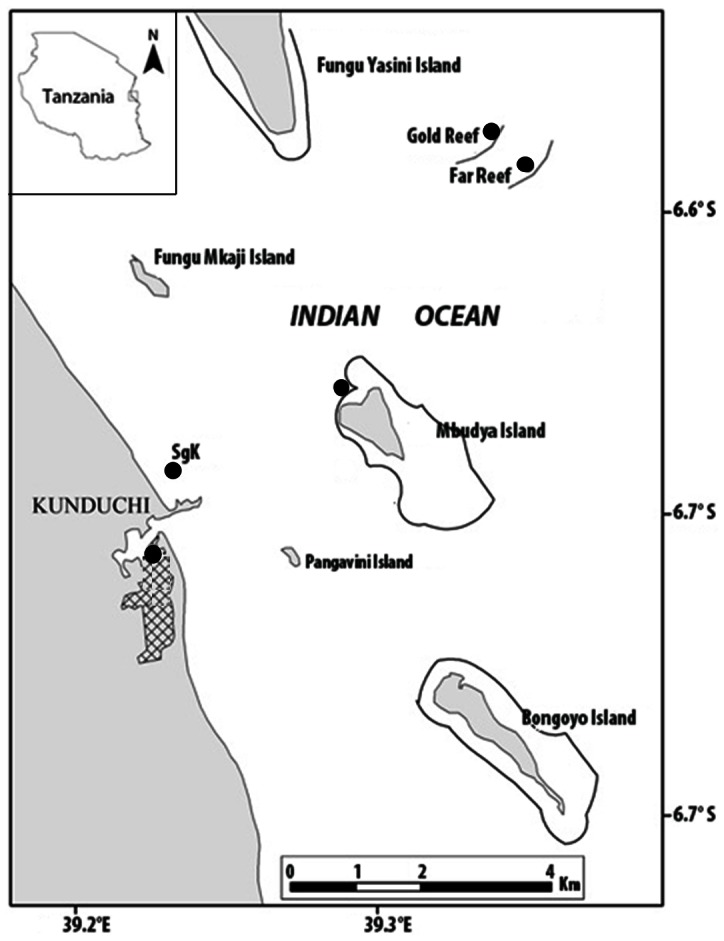
Map of the study area. Reef contours (approx. 17 m depth) are indicated by thick black lines. Hatched area indicates location of the mangrove forest. SGK  =  sampling site at the shoreline seagrass bed at Kunduchi. Nearshore reefs fringe the island of Mbudya, while offshore reefs are located at ‘Far Reef’ and ‘Gold Reef’.

The Kunduchi area in Dar es Salaam Tanzania, where the study was conducted, has only one mangrove-lined creek (Manyema Creek), an extensive shoreline seagrass bed, two nearshore islands with fringing coral reefs which are separated from the mainland by a 15 m deep channel running almost parallel to the coastline, and several offshore submerged deep coral reefs (Figure 1). The mangroves are dominated by *Sonneratia alba* along the sides of the Manyema creek. The creek receives substantial freshwater input only during heavy rainfall. The seagrass bed along the shoreline of the mainland lies at 0.5–5 m depth and the nearshore reefs along the island of Mbudya lie at 2–4 m depth, depending on the tides, while the deep offshore coral reefs are located at 15–20 m followed by mudflats at greater depths [40].

Three fish species, vi*z*. *Lethrinus harak*, *L. lentjan*, and *Lutjanus fulviflamma*, were selected for this study. Studies based on size-frequency data suggest that these species undergo ontogenetic mangrove/seagrass-to-reef habitat shifts [40], [41], [44], [45]. Fishes were collected from three different habitats (mangrove, seagrass, coral reef) (Table 1). An earlier visual census study showed that these habitats and locations harbored highest densities of the selected species ([40], Kimirei unpubl. data); nevertheless it is possible that we did not sample some minor juvenile habitat types in the area. Adults of *L. lentjan* and *L. fulviflamma* were caught from both the nearshore reef around Mbudya Island (about 3 km to mangrove) and two offshore reefs (∼9 km to mangrove), whereas *L. harak* could only be caught from the nearshore reef due to their very low abundances on the offshore reefs [40]. Fishes from the mangrove habitat were collected with a 1×10 m seine net which was dragged against the current during outgoing tide, as well as using hook and line. Fishes from the seagrass beds were all purchased from local fishermen that utilized beach seines at low tide. As fishermen operated their seine nets in front of the research institute the origin of these fish could be confirmed. Specimens from the coral reef were caught using a spear gun. All specimens were measured for total length (TL) and weight before the sagittae otoliths were removed, cleaned and stored pending analysis.

**Table 1:** The number of individuals collected per habitat and species per year.

	*Lethrinus harak*				*Lethrinus lentjan*				*Lutjanus fulviflamma*			
Year	NCR	OCR	SG	MG	NCR	OCR	SG	MG	NCR	OCR	SG	MG
2007	-	-	5	-	-	-	-	-	-	-	17	16
2008	1	-	3	14	1	-	13	4	-	-	7	6
2009	25	-	28	-	1	-	6	-	-	-	-	-
2010	-	-	-	-	56	-	-	-	-	22	-	-
2011	-	-	-	-	-	-	-	-	20	-	-	-
2012	-	-	-	-	-	20	-	-	-	-	-	-
Size range (cm)	24.6–39.6	-	8.2–25.2	3.3–10.7	21.2–35.6	16.1–38.4	8.0–20.4	3.8–9.0	17.9–22.3	16.2–20.0	14.4–20.9	4.0–13.2

NCR  =  nearshore coral reefs; OCR  =  offshore coral reefs; SG  =  seagrass bed; MG  =  mangroves; size range  =  total fish length.

### Otolith Analysis

After cleaning with deionized water, otoliths were mounted on glass plates and embedded in Araldite resin. The embedded otoliths were then cross-sectioned in the transverse plane through the core. For juvenile fishes from the mangroves and seagrass bed the outer otolith margin which reflects the current habitat was analyzed. For larger fishes from the reef, both the juvenile zone (which is the area directly adjacent to the core) which reflects earlier life in putative nurseries, and the outer otolith margin reflecting the adult reef habitat, were analyzed. The location for sampling the juvenile zone in adult otoliths was based on the mean otolith width of the mangrove/seagrass juvenile fish. For large *L. harak* (≥15 cm TL) from the seagrass beds, both the inner and outer otolith zones were analyzed to additionally determine the degree to which large juveniles from seagrass beds had spent their earlier juvenile stage in mangrove habitat and had moved to seagrass beds afterwards.

The sectioned otoliths were drilled with a micromill that produced otolith CaCO_3_ powder from a crater with a diameter of approximately 0.35 mm. Two craters were drilled per sample on opposite sides of the cross section to provide enough otolith powder for analysis. The powder (weight ≥10 µg) was collected with a scalpel and put into a glass tubes for further analysis. A few drops of pure (100%) orthophosphoric acid were added to the tube containing the powder at 80°C to dissolve all CaCO_3_. The isotope ratios of ^12^C/^13^C and ^16^O/^18^O of the released CO_2_ were measured using a Gas Bench mass spectrometer equipped with an automated carbonate extraction line (Kiel device). The NIST SRM 8544 (NBS 19) was used as a carbonate standard, which was routinely monitored during sample runs. The precision of analyses based on the measurements of this standard was within 0.05‰.

### Data and Statistical Analysis

**Figure 2:**
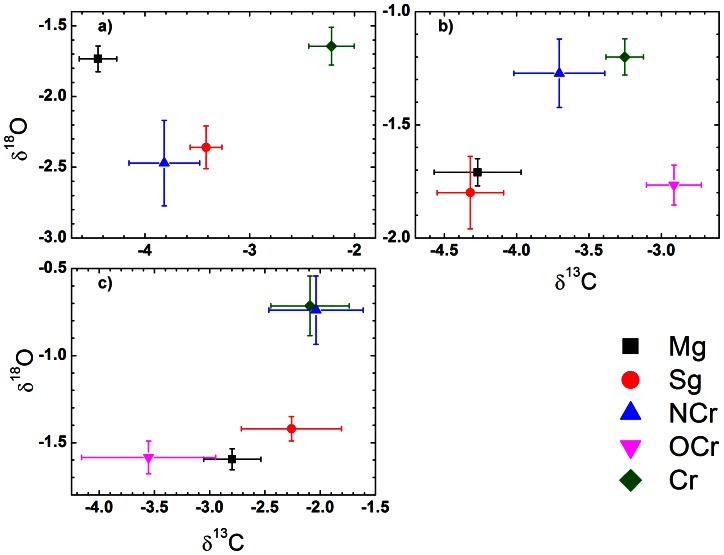
Mean (± SE) otolith δ^13^C and δ^18^O from the outer otolith margins of juvenile fish collected from mangroves (Mg) and seagrass (Sg) habitats, from the inner (juvenile) parts of otolith of adult fish collected from nearshore (NCr) and offshore (OCr) reefs, and from the outer otolith margins of coral reef adults (Cr), averaged per habitat for each of the species: a) *Lethrinus harak*, b) *Lethrinus lentjan*, and c) *Lutjanus fulviflamma*.

Combined stable carbon (δ^13^C) and oxygen (δ^18^O) signatures of the juvenile (inner) otolith sections from adult reef fish were compared to that of the outer otolith section from juvenile fish living in mangrove or seagrass habitats to determine whether adult fish had passed through either of these juvenile habitats. Fishes caught from the coral reef spanned a wide size range (Table 1), thus reflecting different birth years. We therefore collected juvenile fish from the putative mangrove and seagrass nurseries in different years, to incorporate into our analysis potential temporal variability in otolith stable isotope signatures within nursery habitats. A MANOVA showed that either δ^13^C or δ^18^O varied significantly (F>6.28, p<0.001) across years for the 3 species, but never both stable isotope signatures together, meaning that habitats could always be distinguished throughout time based on at least one stable isotope signature. Notwithstanding some temporal variability, there was little overlap among habitat signatures (see Fig. 2). A one-way ANOVA was used to test for differences in otolith δ^13^C and δ^18^O, respectively, between habitats. We tested for significant differences in otolith δ^13^C and δ^18^O between the juvenile signatures of mangrove and seagrass fish, and the adult signatures of coral reef fish (i.e. outer otolith margin). A Gabriel post-hoc test was used for comparison of means, while a Games-Howell post-hoc test was used when the requirement for homogeneity of variance was violated. Significance levels of p<0.05 were used in all tests. A quadratic discriminant function analysis (QDFA) using the jack-knife classification was done to examine classification success of assigning individuals to their known origin. SPSS 20 for Windows was used for all analyses [46].

A maximum likelihood analysis (MLA; estimator # 5) ‘HISEA’ developed by Millar [47] was used to determine the proportion of adult fish originating from the different habitats. For this analysis we used the combination of stable carbon and oxygen isotope signatures of the outer otolith margins of juvenile mangrove and seagrass fish and of adult coral reef fish as baseline data. We used the otoliths of adult reef fish instead of juvenile reef fish as the latter were not observed on the reef during visual surveys. Because otolith composition results from a complex interaction between physiological and environmental factors [14], a potential ontogenetic effect on δ^13^C and δ^18^O cannot be completely ruled out *a priori*. However, we performed linear regression analyses between reef fish body size and stable carbon and oxygen isotope values, respectively, to ascertain that the coral reef signature from the adult otolith margins was reflective of fish from the ocean environment, and did not differ from that of juvenile reef fish (if they were to be found on the reef) due to growth. We tested reef fish between 16 and 40 cm in length, comprising fish from a wide range in age (between ∼3 and 28 years). None of the regressions for either carbon or oxygen showed a significant relationship with size (p>0.11, R^2^<0.14) for any species, except for δ^13^C in *Lethrinus lentjan* which showed a positive relationship (p = 0.01) but with a low R^2^ explaining only a small proportion of the overall variability (R^2^ = 0.32 and 0.12 for nearshore vs. offshore reef fish, respectively). The observation that increase in body size has little to no effect on stable isotope signatures in our fish was further supported by lack of such a relationship in the fishes collected from the seagrass beds as well, which had a sufficiently large size range to test for this potential ontogenetic effect (all separate regression for fish size vs. δ^13^C and δ^18^O, respectively, for all 3 species: p>0.14, R^2^<0.08). These results show that potential ontogenetic effects are minor compared to habitat differences. Signatures from the inner (juvenile) part of adult reef fish otoliths were used in the MLA as the unknown mixed dataset to estimate the origin of these fishes. Finally, to determine in which habitat large juvenile *L. harak*, collected in seagrass habitat, had spent their earlier juvenile stage, juvenile seagrass and mangrove signatures were used as the baseline, and adult outer margins as unknown mixed sample. Maximum likelihood estimates and standard deviations were generated in HISEA by bootstrapping with 500 simulations.

## Results

The three habitats (mangrove, seagrass, and coral reef) differed significantly based on otolith δ^13^C and/or δ^18^O signatures (Table 2, Fig. 2). For *L. harak*, otolith δ^13^C differed significantly among all habitats, while seagrass δ^18^O signatures differed from that of the mangrove and coral reef, respectively. For *L. lentjan* both otolith δ^13^C and δ^18^O differed between the coral reef and seagrass, but not between the coral reef and mangroves, or between seagrass and mangroves. When seagrass and mangrove signatures were combined they did significantly differ from the coral reef signature. For *L. fulviflamma* only otolith δ^18^O differed among all habitats. Classification success based on a quadratic discriminant function analysis was very high for *L. harak* and *L. lentjan* (>73%) and relatively high for *L. fulviflamma* (61%) (Table 3). The classification was based on δ^13^C as well as δ^18^O otolith signatures and both isotope ratios were important in discrimination of the three different habitats.

**Table 2:** Results of a one-way ANOVA on otolith δ^13^C and δ^18^O, respectively, of *Lethrinus harak*, *Lethrinus lentjan*, and *Lutjanus fulviflamma* for three potential juvenile habitats.

	δ^13^C						δ^18^O					
			post-hoc						post-hoc			
			CR	CR	MG	CR			CR	CR	MG	CR
			vs	vs	vs	vs			vs	vs	vs	vs
	F	p	SG	MG	SG	SG+MG	F	p	SG	MG	SG	SG+MG
***Lethrinus harak***	27.335	<0.001	<0.001	<0.001	0.002		7.741	0.001	0.002	**0.845**	0.002	
***Lethrinus lentjan***	6.351	0.003	0.003	**0.115**	**0.999**	<0.001	6.666	0.002	0.001	**0.240**	**0.989**	<0.001
***Lutjanus fulviflamma***	1.155	0.320	**0.947**	**0.572**	**0.371**		11.039	<0.001	0.003	<0.001	0.011	

Non-significant values (p>0.05), which indicate no differences among habitats, are indicated in bold. Due to non-significant post-hoc tests for otolith δ^13^C and δ^18^O of *L. lentjan* between mangrove and seagrass these two habitats had to be combined. CR  =  coral reef; SG  =  seagrass bed; MG  =  mangroves.

**Table 3:** Estimated contribution (% ± SD) from Maximum Likelihood Analysis of different potential juvenile habitats to adult nearshore and offshore reef populations of three reef fish species, and large juvenile (>15 cm TL) *L. harak* in seagrass beds.

				Juvenile habitat			
	N	Mean density (100 m^−2^)	Classification success (%)	SG	MG	CR	SG+MG
***Lethrinus harak***							
Nearshore reef fish	25	0.03±0.02	73.3	52.9±18.6	28.9±13.6	18.2±14.1	
Large seagrass fish	9	0.21±0.18	82.0	69.7±20.0	30.3±20.0	-	
***Lethrinus lentjan***							
Nearshore reef fish	20	0.18±0.05	77.4			65.3±22.0	34.7±22.0
Offshore reef fish	54	0.07±0.11				72.4±12.2	27.6±12.2
***Lutjanus fulviflamma***							
Nearshore reef fish	19	0.09±0.07	60.5	18.9±21.2	0.03±0.6	81.1±21.2	
Offshore reef fish	21	1.25±1.45		1.2±6.1	98.9±6.2	0.0±0.0	

Classification success is based on a quadratic discriminant function analysis using jack-knife classification to examine the success of assigning individuals to their known origin. Both analyses are based on δ^13^C and δ^18^O values combined. Because seagrass and mangrove habitat signatures did not differ for *L. lentjan*, they had to be combined for this analysis. CR  =  coral reef; SG  =  seagrass bed; MG  =  mangroves.

For all three species, except *L. fulviflamma* from the nearshore reef, the δ^13^C and/or δ^18^O signatures from juvenile margins of nearshore and offshore reef fish individuals were significantly different compared to the adult margin signatures (Fig. 2), suggesting that the two life stages used different habitats. The results of the maximum likelihood analysis using both carbon and oxygen stable isotopes showed that adults from the three species had passed through different juvenile habitats. Adult *L. harak* fish from the reef were only collected on nearshore reefs. Over 81% of these fish originated from either mangrove (29%) or seagrass (53%) habitats, while the remainder (18%) had grown up on the reef (Table 3). Large specimens caught on the seagrass bed originated largely from the seagrass (70%) and partly from the mangrove (30%) habitat. Most adults of *L. lentjan* that were collected on the offshore reefs had spent their juvenile stage on coral reefs (72%) compared to a much smaller amount of fish that had passed through seagrass and mangrove habitats (28%). Nearshore adult reef fish of this species also predominantly originated from coral reefs (65%). *L. fulviflamma* showed contrasting results: for adults collected on offshore reefs, mangroves were the dominant juvenile habitat (99%) and none of these fish had a juvenile signature that indicated a contribution from the coral reef. In contrast, nearshore *L. fulviflamma* adults mainly showed a coral reef signature (81%) in the juvenile zones of their otoliths, compared to that from seagrass (19%) or mangrove (0.03%) habitat. However, *L. fulviflamma* densities on nearshore reefs were at least an order of a magnitude smaller compared to those on offshore reefs (Table 3).

## Discussion

This study is one of the first to present conclusive evidence that mangrove and seagrass habitats replenish reef fish populations in an Indo-Pacific locality, and is the first to identify the relative importance of multiple potential juvenile habitats in replenishing adult populations. The importance of these putative juvenile habitats differed among fish species, and among reefs located at different distances from these habitats. For adults collected on nearshore reefs, the combined contribution of mangrove and seagrass habitat was highest for *L. harak* (82%), followed by *L. lentjan* (35%) and *L. fulviflamma* (19%), while for offshore reefs this was 28% and 100% for the latter two species, respectively. The large contribution of coral reefs to the nearshore as well as offshore adult populations of *L. lentjan* (65–72%) suggests that *L. lentjan* populations may be largely self-replenishing by the reef habitat. In contrast, the juvenile source habitats that were important for *L. fulviflamma* showed a large contrast for adults collected from nearshore vs. offshore reefs. However, considering that reef fish densities for this species were 13 times higher on offshore than nearshore reefs (1.25 vs. 0.09 fish per 100 m^2^, respectively), most of the reef fish in this coastal area had likely originated from mangroves in terms of total population size. The observed variability in juvenile habitat contribution to adult populations indicates clear species-specific differences in nursery habitat dependency as well as presence of spatial differences in degree of population replenishment by nursery habitats [40].

The results from our otolith study supported those from field surveys for some species, but not for others. Adult populations of *L. harak* were replenished to a greater degree by seagrass beds than by mangroves. This result corresponds to previous visual census data which also showed that mangroves are less important as a juvenile habitat for *L. harak* compared to seagrass [40]. On the contrary, *L. lentjan* showed a higher contribution from the coral reef to both nearshore and offshore adult populations than seagrass bed and mangrove combined, whereas visual census surveys suggest that this species primarily uses seagrass beds as juvenile habitats [40]. The above indicates that visual census data should be interpreted with caution, and be combined with data collected by different methods to effectively identify and assess the nursery function of putative juvenile habitats. Clearly, juvenile fish densities as obtained through visual surveys only reflect the standing stock at the time of observation. They fail to quantify, however, how much of this standing stock will continue to survive and move to reefs at some point in time. Differential mortality among juvenile habitats, for example, could subsequently lead to different population contributions by individual habitats as would be deduced from absolute abundances.

Because of the temporary nature of the Indo-Pacific mangroves' availability to fishes due to the tidal regime, and the relatively low number of species using them as juvenile habitats [29], [34], [36], [48], their nursery function has long been questioned (see [11], [48]). The permanent inundation of Caribbean mangroves, on the other hand, translates to high abundances of juvenile coral reef fishes that use these habitats [49]. Nevertheless, the range in degree of replenishment of reef populations by recruits from mangrove habitats does not seem to be very different between the two regions – e.g. Indo-Pacific: 88% for *Lutjanus fulvus* ([17]; based only on mangrove-reef migrations over short time scales as stable isotope analysis of muscle tissue was used); 29% and 99% for *Lethrinus harak* and *Lutjanus fulviflamma*, respectively (this study) vs. Caribbean: 36% for *Haemulon flavolineatum* [15]; 40–74% for *Haemulon flavolineatum* and 99% for *Lutjanus apodus* [20]. This indicates that Indo-Pacific mangroves can play an equally important role as juvenile habitat for some species as is the case in the Caribbean.

Using three potential juvenile habitats (mangrove, seagrass, coral reef) as a source, the current study suggests that no single habitat maintains reef fish populations of these species (except perhaps *L. fulviflamma* on offshore reefs), but act together in replenishing adult populations. Previous studies have shown high (88–99%) contributions from single juvenile habitats to adult reef fish populations, but none of these studies included multiple juvenile habitats, or the coral reef itself was not taken into account as a possible juvenile habitat [17], [20], [22]. Studies showing single habitats that contribute close to 100% of recruits to adult populations are rare in general [15], [22], [50], [51]. The different contributions of the three habitats in the present study indicates that maintenance of reef fish populations in the Indo-Pacific depends on habitat mosaics rather than on individual habitats [10], [52], which is an important consideration for species and fisheries management and conservation.

The usage of coastal habitat mosaics by juveniles of some reef fish could provide an insurance effect (e.g. [53]) against natural or human perturbations, or failure of management efforts in single habitats. Coastal habitats are currently under high pressure from anthropogenic activities and climate change [4], [5], [6], [54], [55] which are threatening their existence, and pose a threat to the recruitment, persistence and sustainability of marine fish populations. Coral reefs are being seriously overfished across the globe [56]. In this light, contributions of juvenile habitats with a currently lower than average contribution to adult populations could become critical in maintaining offshore fish stocks at sustainable levels. Identification of nursery habitats has traditionally been based on habitats that supply a higher number of recruits to adult populations than the average across all juvenile habitats [12], [57]. However, without any guarantees that the most productive nursery habitat can be managed effectively or guarded against natural disturbances, we should spread the risk of potential management failure by conserving seascapes containing multiple patches of connected habitats. This approach also incorporates multiple juvenile habitat usage throughout ontogeny as well as maintenance of habitat linkages resulting from daily feeding or shelter-seeking migrations [58], [59], [60]. Our study showed that about a third of the large juvenile *Lethrinus harak* in seagrass beds had utilized mangroves during their earlier life stage. Such niche shifts are very common in tropical coastal fish species [61], [62], and effective replenishment of adult populations can only be accomplished by incorporating all habitats that are successively used by fishes during their juvenile stage.

In conclusion, our results provide strong support for a nursery role of Indo-Pacific mangroves for certain species of reef fishes, but also indicate that seascape structure plays a vital role, and habitat mosaics rather than individual habitats should be conserved to maintain effective replenishment of offshore fish stocks.
